# Immune Checkpoint Blockade: A New Paradigm in Treating Advanced Cancer

**DOI:** 10.6004/jadpro.2014.5.6.3

**Published:** 2014-11-01

**Authors:** Kristen M. Kreamer

**Affiliations:** Fox Chase Cancer Center, Philadelphia, Pennsylvania

## Abstract

The approval of the immune checkpoint inhibitor ipilimumab for the treatment of advanced melanoma in 2011 spearheaded the development of other anticancer therapies with immune mechanisms of action, including other immune checkpoint inhibitors. Instead of acting directly on the tumor, these therapies work to "remove the brakes" on the immune system to restore antitumor immune responses. In addition to ipilimumab, which targets the cytotoxic T lymphocyte-associated antigen 4 pathway, several new drugs that target the programmed death-1 pathway are in phase III trials across tumor types, including melanoma, lung cancer, and renal cell carcinoma. In keeping with their unique mechanism of action, these immune checkpoint inhibitors have shown both conventional and unconventional response patterns, including initial apparent tumor progression followed by regression, and adverse events (AEs) that are likely immune-related. Advanced practitioners (APs) treating patients receiving immuno-oncology agents are in a key position to educate patients about expectations with these therapies and to screen patients for AEs and initiate appropriate and timely interventions. This review summarizes current immune checkpoint inhibitor data and provides patient management strategies for APs to optimize patient outcomes with these novel therapies.

The ability of the immune system to detect and eliminate cancer was first proposed over 100 years ago (Cann, van Netten, & van Netten, 2003). Since then, T cells reactive against tumor-associated antigens have been detected in the blood of patients with many different types of cancers, suggesting a role for the immune system in fighting cancer (Nagorsen, Scheibenbogen, Marincola, Letsch, & Keilholz, 2003). However, tumors can escape host immunity by manipulating the tumor microenvironment and driving immunosuppression (Kim, Emi, & Tanabe, 2007), meaning that patients cannot mount a potent enough immune response to fully eliminate cancer cells.

The goal of immunotherapy is to restore or augment antitumor immune responses, and the objective responses seen with vaccination and other immune-based strategies support this approach (Kantoff et al., 2010; Hodi et al., 2010; Topalian et al., 2012). An increased understanding of tumor immunology has led to the identification of novel targets for new immune-based approaches, including a group of cell-surface molecules known as *immune checkpoint proteins* (Pardoll, 2012).

In 2011, ipilimumab (Yervoy) became the first immune checkpoint inhibitor to be approved by the US Food and Drug Administration specifically for the treatment of unresectable or metastatic melanoma (National Comprehensive Cancer Network, 2014). The clinical success of this agent has reenergized scientific investigation into the blockade of other immune checkpoints, as well as into the evaluation of these agents in cancers not traditionally considered "immunogenic," such as lung cancer.

Immune checkpoint blockade therapies differ from traditional therapies not only in their mechanisms of action, but also in their response patterns and adverse event (AE) profiles. As immunotherapies become available for an increasing number of cancer types, it will be important for advanced practitioners (APs) to understand the basic differences from standard chemotherapies so as to effectively evaluate responses, manage side effects, and educate patients and other health-care partners.

## MECHANISM OF ACTION OF IMMUNE CHECKPOINT BLOCKADE THERAPIES

Standard chemotherapies act directly on cancer cells to inhibit tumor growth or cause tumor cell death (Cepeda et al., 2007; Florea & Büsselberg, 2011). Common mechanisms of action for chemotherapeutic agents include interrupting DNA synthesis, interrupting DNA replication and repair, and inhibiting cell division—all of which inhibit cell growth and division processes and trigger natural cell death pathways—both in tumor cells and in normally dividing cells (Cepeda et al., 2007; Hanna et al., 2004; Lyseng-Williamson & Fenton, 2005). Common side effects of cytotoxic therapies (anemia, hair loss, and gastrointestinal symptoms) are likely a result of this mechanism of action.

The targeted therapies erlotinib (Tarceva), afatinib (Gilotrif), crizotinib (Xalkori), and ceritinib (Zykadia) inactivate mutated proteins in tumor cells that drive tumor growth, and bevacizumab (Avastin) inhibits angiogenesis, which limits the tumor’s blood supply, restricting its growth (Sechler et al., 2013). These agents are not cytotoxic, and their side effects differ from those of chemotherapies.

In contrast, immunotherapies act by stimulating the immune system to eliminate cancer cells through natural immune-mediated cell-killing processes. One approach that has shown efficacy in melanoma is blockade of an immune checkpoint pathway (Hoos et al., 2010). Immune checkpoints are receptor:ligand systems on immune cells; when engaged, these cells down-modulate immune responses to prevent autoimmunity and/or to minimize damage to healthy tissue during an immune response (Pardoll, 2012).

The two immune checkpoint pathways that are best understood are the cytotoxic T lymphocyte-associated antigen 4 (CTLA-4) and programmed death-1 (PD-1) pathways, although several others have also been described (Pardoll, 2012). Blockade of any of the inhibitory checkpoint pathways could enhance preexistent antitumor immunity. The different pathways appear to have nonredundant roles, and preclinical and emerging clinical data indicate that blockade of multiple checkpoints may be synergistic (Okudaira et al., 2009; Selby et al., 2013; Wolchok et al., 2013).

**CTLA-4**

T-cell activation is a highly regulated process. To initiate T-cell activation, proliferation, and antitumor effects, the T cell must receive two different signals: (1) T-cell recognition of a presented tumor antigen and (2) a costimulatory signal that strengthens the activation response (Hoos et al., 2010). After a T cell recognizes a tumor antigen, signaling through the CTLA-4 pathway prevents the costimulatory signal, and it serves as a natural inhibitory mechanism on the immune response.

**Figure 1:**
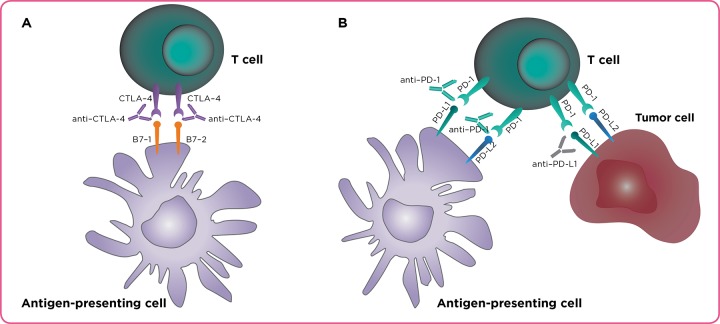
Ipilimumab is a fully human anti–CTLA-4 antibody designed to block CTLA-4:B7 binding, thereby allowing costimulatory signaling and generation of antitumor T-cell responses (A). Anti–PD-1 and anti–PD-L1 monoclonal antibodies work by inhibiting PD-1:PD-L1 binding and restoring antitumor immune responses (B). Adapted with permission from Langer (2014), Lippincott Williams & Wilkins/ Wolters Kluwer Health.

Ipilimumab is a fully human anti–CTLA-4 antibody designed to block CTLA-4 signaling, thereby allowing costimulatory signaling and generation of antitumor T-cell responses (Figure 1). On the basis of improved survival over controls in two randomized trials in unresectable or metastatic melanoma (Hodi et al., 2010; Robert et al., 2011), ipilimumab was approved in the United States (NCCN, 2014) and in many other countries worldwide as therapy for advanced melanoma (specific indications vary by country).

**PD-1/PD-L1**

PD-1 is another inhibitory receptor that is expressed on T cells, but it has a nonoverlapping function from that of CTLA-4. In the cancer setting, the ligands for PD-1, PD-L1 (thought to be the predominant ligand) and PD-L2, are expressed in the tumor microenvironment (Pardoll, 2012; Nirschl & Drake, 2013; Zou & Chen, 2008). PD-1:PD-L1 ligand binding leads to inhibition of T-cell proliferation and decreased production of inflammatory cytokines (Okazaki & Honjo, 2006; Peng et al., 2012).

Various anti–PD-1 and anti–PD-L1 monoclonal antibodies are currently in advanced stages of clinical development. They include nivolumab (anti–PD-1), pembrolizumab (Keytruda, MK-3475; anti–PD-1), pidilizumab (CT-011; anti–PD-1), MPDL3280A (anti–PD-L1), and MEDI4736 (anti–PD-L1). These agents work by inhibiting PD-1:PD-L1 binding and restoring antitumor immune responses (Table; Figure 1; Langer, 2014). After acceptance of this manuscript for publication, pembrolizumab was approved in September 2014 for the treatment of patients with unresectable or metastatic melanoma with disease progression following ipilimumab and, if *BRAF^V600^* mutant, a BRAF inhibitor. Very little clinical data were available for MEDI4736 at the time of writing, so it is not discussed further in this article.

**Table 1:**
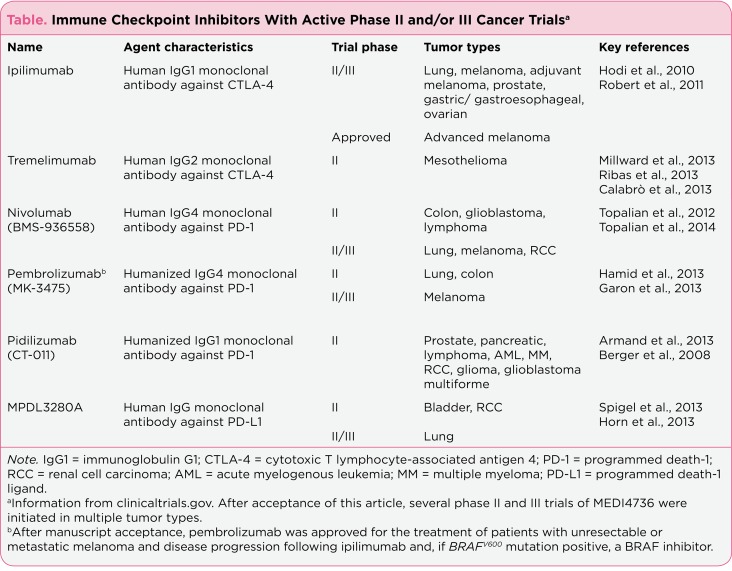
Immune Checkpoint Inhibitors With Active Phase II and/or III Cancer Trials

## EFFICACY OF CTLA-4 INHIBITORS

**Ipilimumab**

*Melanoma*: Ipilimumab, an antibody against CTLA-4, was the first agent to improve median overall survival (OS) in patients with advanced melanoma (Hodi et al., 2010; Robert et al., 2011). The approval of ipilimumab was supported by a phase III, placebo-controlled, randomized trial of previously treated patients who received ipilimumab monotherapy, ipilimumab plus a peptide vaccine, or vaccine alone (Hodi et al., 2010). Patients who received ipilimumab monotherapy (3 mg/kg every 3 weeks for up to 4 doses) had a median OS of 10.1 months, and an OS rate of 46% and 24% at 1 year and 2 years, respectively. The addition of glycoprotein (gp100) vaccine did not significantly improve the benefit of ipilimumab therapy. The mean OS in the control group receiving gp100 alone was 6.4 months (*p* = .003 vs. ipilimumab monotherapy). A second randomized phase III trial demonstrated significantly longer OS for patients with previously untreated metastatic melanoma who received chemotherapy plus ipilimumab over chemotherapy alone (Robert et al., 2011).

*Lung Cancer*: Historically, lung cancer has not been considered a tumor responsive to immunotherapy. However, ipilimumab plus chemotherapy was thought to be a rational approach to lung cancer, as preclinical studies have shown that chemotherapy can cause tumor antigen release, promote T-cell–mediated antitumor responses, and synergize with anti–CTLA-4 antibody treatment (Jure-Kunkel et al., 2013; Zitvogel, Galluzzi, Smyh, & Kroemer, 2014).

Therefore, ipilimumab in combination with paclitaxel and carboplatin was evaluated as a first-line therapy in patients with extensive-stage small cell lung cancer (SCLC) and in patients with non–small cell lung cancer (NSCLC) in one phase II study. In both patient subgroups, a phased regimen of ipilimumab given after two doses of paclitaxel/carboplatin showed potential survival improvements over paclitaxel/carboplatin plus placebo. For SCLC patients, the median OS was 12.9 months with phased ipilimumab vs. 9.9 months for the chemotherapy/placebo group (*p* = .13; Reck et al., 2013). For NCSLC patients, the median OS was 12.2 months with phased ipilimumab vs. 8.3 months with chemotherapy/placebo (p = .23; Lynch et al., 2012).

Of note, unlike with the phased regimen, efficacy improvements were not seen with a concurrent ipilimumab plus chemotherapy regimen as compared with the chemotherapy plus placebo control group. The investigators speculated that exposure to chemotherapy prior to ipilimumab may have led to enhanced activation of T cells, similar to that seen in preclinical models (Lynch et al., 2012; Reck et al., 2013). As further studies are needed to validate this initial evidence of activity, phase III trials of ipilimumab following chemotherapy are currently underway in patients with SCLC and NSCLC (www.clinicaltrials.gov).

*Prostate Cancer*: Radiotherapy in combination with anti-CTLA therapy has also shown synergistic antitumor effects in preclinical models and clinical reports (Demaria et al., 2005; Postow et al., 2012). In a phase I/II trial in patients with metastatic castration-resistant prostate cancer, ipilimumab plus radiotherapy showed evidence of activity. Of the 50 patients receiving ipilimumab (10 mg/kg) plus radiotherapy (8 Gy/lesion), 8 patients had a 50% decline in prostate-specific antigen levels, 1 patient had a complete response (CR), and 6 patients had stable disease (Slovin et al., 2013). Clinical trials of ipilimumab are ongoing in metastatic prostate cancer, including as a single agent vs. placebo (phase III) and in combination with sipuleucel-T (Provenge, phase II). Ipilimu-mab in combination with radiotherapy is also being investigated in patients with metastatic melanoma (Table; www.clinicaltrials.gov).

**Tremelimumab**

Tremelimumab, also an antibody against CTLA-4, has been evaluated in multiple tumor types (Calabrò, Danielli, Sigalotti, & Maio, 2010; Tarhini, 2013). Ipilimumab is an immunoglobulin G1 (IgG1) antibody and tremelimumab is an immunoglobulin G2 (IgG2) antibody (Table), which could account for the differences in clinical activity between the two agents (Tarhini, 2013).

*Melanoma, Breast Cancer, and Mesothelioma*: Despite initial evidence of activity against melanoma in earlier trials, a phase III trial of tremelimumab in patients with advanced melanoma did not meet its primary endpoint, possibly due to study design issues (Ribas et al., 2013; Tarhini, 2013). In a small phase I study, 11 patients with advanced, hormone-responsive breast cancer receiving tremelimumab plus exemestane had stable disease for 12 weeks (Vonderheide et al., 2010). Based on encouraging results of a phase II trial in patients with previously treated malignant mesothelioma (disease control in 31% of patients, median progression-free survival of 6.2 months, and median OS of 10.7 months; Calabrò et al., 2013), phase III trials of tremelimumab in malignant mesothelioma have been initiated (Table; www.clinicaltrials.gov).

## EFFICACY OF PD-1/PD-L1 INHIBITORS

Several anti–PD-1 and anti–PD-L1 antibodies in clinical development have shown promising activity in cancer studies. Those with ongoing phase II or III trials in patients with cancer include the anti–PD-1 agents nivolumab, pembrolizumab (MK-3475), and pidilizumab (CT-011) and the anti–PD-L1 agent MPDL3280A (Table). Although only phase I data are available at the time of writing, it appears that these therapies may be associated with higher response rates, shorter times to response, and more favorable safety profiles as compared with anti–CTLA-4 antibodies. However, as data from head-to-head clinical trials are not yet available, it is unclear whether these apparent differences are due to targeting the PD-1 vs. CTLA-4 pathway or to clinical trial patient populations and/or other factors.

Clinical studies are actively investigating whether tumor expression of PD-L1 can serve as a biomarker for patients more likely to respond to PD-1 pathway inhibitors. To date, some studies have shown higher response rates in patients with tumors expressing intermediate or high levels of PD-L1 as compared with tumors with low or negative PD-L1 expression (Antonia et al., 2013; Garon et al., 2013; Horn et al., 2013; Topalian et al., 2012; Weber et al., 2013). However, responses have also been seen in patients with low or undetectable levels of PD-L1 (Antonia et al., 2013; Garon et al., 2013; Weber et al., 2013). Some ongoing trials of PD-1/PD-L1 inhibitors are prospectively enrolling only patients with PD-L1–positive tumors. Other trials are assessing tumor PD-L1 expression at baseline and will report data comparing outcomes based on PD-L1 positive or negative expression (www.clinicaltrials.gov).

**Nivolumab**

A phase I nivolumab dose-escalating (0.1 to 10 mg/kg every 2 weeks) study initially reported the results of 296 patients with metastatic melanoma, NSCLC, colorectal cancer (CRC), castration-resistant prostate cancer, or renal cell carcinoma (RCC; Topalian et al., 2012). Nivolumab produced objective responses in a substantial portion of patients with melanoma, NSCLC, and RCC. In contrast, no objective responses were observed in patients with CRC or prostate cancer, although patient numbers were considerably smaller for these two tumor types (Topalian et al., 2012).

*NSCLC*: After a longer follow-up period for 129 patients with NSCLC who received higher dosing, objective response rates by nivolumab dose were 3% (1 mg/kg), 24% (3 mg/kg), and 20% (10 mg/kg) by Response Evaluation Criteria in Solid Tumors (RECIST) v1.0 (Brahmer et al., 2013). All patients had been previously treated, and 54% had received three or more prior therapies. Objective responses were observed in patients with both squamous and nonsquamous NSCLC, and survival 42% and 24% at 1 and 2 years, respectively.

*Melanoma*: In 107 patients with melanoma, some with more than 4 years of follow-up, objective response rates ranged from 20% to 41% across doses from 0.1 to 10 mg/kg, and the median duration of response was 2 years. The median survival was 16.8 months, and survival rates were 62% at 1 year and 43% at 2 years (Topalian et al., 2014).

*RCC*: In patients with RCC, objective responses of 28% (1 mg/kg) and 31% (10 mg/kg) were observed, and again, in some patients, these responses lasted for 2 years or longer. One- and two-year survival rates for RCC patients were 70% and 52%, respectively (Drake et al., 2013

Nivolumab has an active clinical development program, with phase II and III trials ongoing in numerous tumor types (Table; www.clinicaltrials.gov).

**Pembrolizumab (MK-3475)**

*Melanoma*: In a phase I study, 135 patients with advanced melanoma who had previously received or not received ipilimumab were administered pembrolizumab at a dose of either 10 mg/kg (every 2 or 3 weeks) or 2 mg/kg (every 3 weeks; Hamid et al., 2013). The overall response rate across all doses was 38% by RECIST v1.1. The cohort receiving the maximum dose (10 mg/kg every 2 weeks; n = 52) had a response rate of 52%, the highest observed in the study. Prior treatment with immunotherapy, including ipilimumab or interleukin-2, did not appear to affect the activity or safety profile of pembrolizumab (Hamid et al., 2013).

*NSCLC*: An ongoing phase I study is administering pembrolizumab (10 mg/kg every 3 weeks) to previously treated patients with NSCLC. In an interim analysis of 38 patients, the objective response rate by RECIST v1.1 was 21%, the median OS was 51 weeks, and the median progression-free survival was 9.7 weeks (Garon et al., 2013).

Pembrolizumab is being further investigated in lung cancer, melanoma, and colon cancer (Table; www.clinicaltrials.gov).

Pi**dilizumab (CT-011)**

*Hematologic Malignancies*: Pidilizumab has been evaluated primarily in hematologic malignancies. In a phase I trial, there was initial evidence of activity across several hematologic malignancies when used as a single agent (Berger et al., 2008). Pidilizumab plus rituximab (Rituxan) in patients with relapsed follicular lymphoma resulted in an objective response rate of 66% (complete, 52%; partial, 14%) in a phase II trial (Westin et al., 2012).

In another phase II trial, pidilizumab was used after an autologous hematopoietic stem cell transplant in patients with B-cell lymphomas. The progression-free survival at 16 months was 72%, and in patients with measurable disease after transplant, the overall response rate with pidilizumab was 51%, and the complete remission rate was 34% (Armand et al., 2013).

In other phase II trials, pidilizumab is being tested in combination with disease-specific vaccines in patients with multiple myeloma, acute myelogenous leukemia, and RCC (www.clinicaltrials.gov). In solid tumors, combinations of pidilizumab plus gemcitabine in resected pancreatic cancer and pidilizumab plus sipuleucel-T and cyclophosphamide in prostate cancer are being evaluated (Table; www.clinicaltrials.gov).

**MPDL3280A**

Unlike nivolumab and pembrolizumab, MPDL3280A is designed to block one specific ligand in the PD-1 pathway—PD-L1—as opposed to blocking the PD-1 receptor.

*NSCLC*: This agent is being assessed in an ongoing dose-ranging phase I study in patients with NSCLC (Horn et al., 2013). Patients receive MPDL3280A (0.03 to 20 mg/kg) every 3 weeks for up to 1 year. An overall response rate of 23% (12 of 53 patients) using RECIST v1.1 criteria has been reported, and patients with both squamous and nonsquamous histologies showed clinical responses. In some patients, rapid tumor shrinkage has been observed, and most responses were ongoing at the time of analysis, with follow-up ranging from approximately 24 to 75 weeks (Horn et al., 2013).

MPDL3280A is the focus of ongoing phase II and III trials in bladder cancer, lung cancer, and RCC (Table; www.clinicaltrials.gov).

## EFFICACY OF COMBINATION IMMUNE CHECKPOINT BLOCKADE

Taken together, the reports described here indicate that targeting the CTLA-4 or PD-1 pathway appears to be promising in the treatment of various forms of cancer. Furthermore, recent studies have focused on evaluating ipilimumab in combination with one of the PD-1 pathway–blocking agents. These combinations have produced rapid and extensive tumor regression, which may exceed responses from CTLA-4 or PD-1/PD-L1 single-agent therapy based on preliminary observations (Wolchok et al., 2013).

*Melanoma*: Wolchok and colleagues (2013) conducted a phase I trial that included 53 patients with advanced melanoma who received concurrent therapy with nivolumab and ipilimumab. The objective response rate was 40%, and evidence of clinical activity (i.e., including stable disease) was observed in 65% of patients—well above the rate previously observed in other studies with either of these agents alone. Of 16 patients who had a tumor reduction of 80% or more at 12 weeks, 5 had a complete response. Among patients who received the maximum doses associated with an acceptable level of AEs (nivolumab at 1 mg/kg and ipilimumab at 3 mg/kg), 9 of 17 patients (53%) had an objective response, with a tumor reduction of 80% or more (including 3 complete responses) at their first scheduled assessment.

Several additional studies evaluating nivolumab plus ipilimumab regimens are ongoing in patients with melanoma, lung cancer, and RCC (www.clinicaltrials.gov). It is not clear whether this level of activity is maintained if the agents are sequenced instead of combined, but again, this question is the subject of several ongoing clinical trials. Additionally, it is as yet unclear whether this activity will differ when ipilimumab is paired with other PD-1/PD-L1–blocking agents.

## RESPONSE PATTERNS TO TREATMENT WITH IMMUNE CHECKPOINT INHIBITORS

Because ipilimumab is the immune checkpoint inhibitor that has undergone the most clinical study, our understanding of how to measure immunotherapeutic efficacy of these agents has been informed primarily by the experience with ipilimumab. Clinical studies with ipilimumab in advanced or metastatic melanoma have shown heterogeneous response patterns, some of which resemble typical responses following chemotherapy, and others that are unusual and different from responses seen with chemotherapy (Wolchok et al., 2009). For example, responses can be delayed for many weeks and may even occur after what may appear as disease progression on a scan (e.g., increase in size or number of lesions); this may create dilemmas for patients and clinicians about whether to continue ipilimumab therapy or proceed to a subsequent therapy.

To date, four response variations have been described: (1) response in baseline (index) lesions similar to that observed after chemotherapy or targeted agents; (2) stable disease, which may or may not be followed by a slow, steady decline in tumor burden; (3) response after an increase in tumor burden; and (4) response in index and new lesions accompanied by the appearance of other new lesions. All four patterns have been associated with favorable survival, but patterns (3) and (4) might be unfamiliar in the clinic.

The novel patterns of response seen with ipilimumab are consistent with its immunologic mechanism of action, which restores the antitumor activity of T cells. Mounting an effective antitumor immune response that leads to tumor regression requires a coordinated effort between T cells and numerous other types of immune cells. This process may be quicker in some patients and slower in others. In fact, it may take some patients many weeks or months to respond to ipilimumab, and such a delay in response, or even evidence of disease progression, does not necessarily herald treatment failure, as it does with chemotherapy (Hoos et al., 2010; Saenger & Wolchok, 2008; Wolchok et al., 2009).

The mechanism of action of ipilimumab suggests not only that there may be a delay in tumor regression, but also that before this happens, the tumor may appear to grow. This could be "true" tumor growth, which occurs before the immune system is activated enough to affect the tumor, or it could be a transient increase in tumor size caused by the infiltration of immune cells into a tumor, which might be mistaken for cancerous growth. For example, in one reported case, an ipilimumab-treated patient had radiologic disease progression; however, since it was accompanied by improvements in constitutional symptoms and lactate dehydrogenase levels, the decision was made to continue the patient on ipilimumab. Shortly after the initial apparent disease progression, the patient had a complete response lasting 1 year (Saenger & Wolchok, 2008).

These unconventional responses have also been seen in some clinical trial patients treated with anti–PD-1 and anti–PD-L1 agents (Topalian et al., 2012; Horn et al., 2013). In routine clinical practice and in clinical trial settings, clinicians should be aware of these potential response patterns when assessing the efficacy of immunotherapies.

## SAFETY PROFILE OF IMMUNOTHERAPY VS. CHEMOTHERAPY

APs are familiar with the side effects commonly associated with cytotoxic therapies such as platinum chemotherapy, which typically include fatigue, nausea, vomiting, and bone marrow suppression. Other important AEs include nephrotoxicity and hearing loss (Florea & Büsselberg, 2011). These toxicities are typically due to "off-target" drug effects on healthy tissue and are usually dose-dependent.

**Figure 2:**
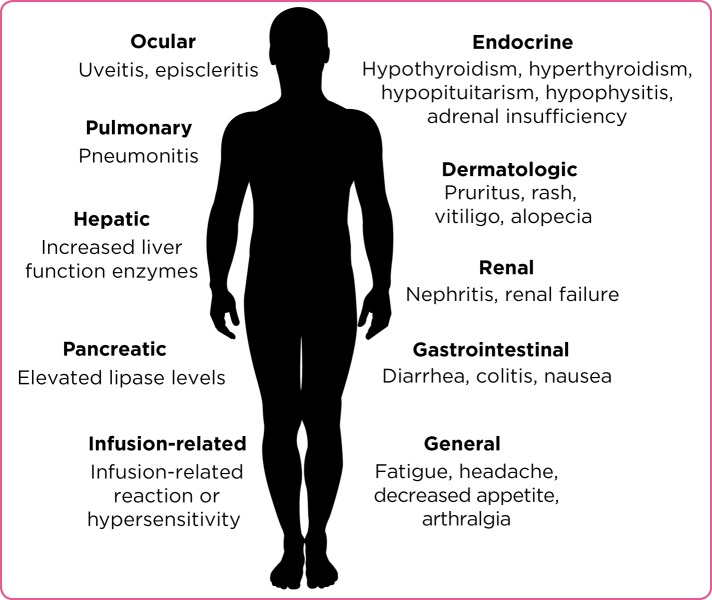
Select immune-related adverse events

Some of the side effects of immuno-oncology agents are also similar to those of conventional agents (i.e., fatigue, nausea, and vomiting), although the underlying etiologies are likely different. This may be a possible explanation for why these AEs with immune checkpoint inhibitors appear to be less common and less severe than those associated with chemotherapy. However, immune checkpoint inhibitors are associated with AEs that have potential immunologic etiologies, which require frequent monitoring and/or unique interventions. Although they are typically manageable by drug discontinuation and/or intervention, these immune-related AEs (irAEs) may be unfamiliar to oncology teams that do not have experience with therapies such as ipilimumab (Figure 2).

To date, most experience with irAEs comes from studies in melanoma patients treated with ipilimumab and early studies of anti–PD-1 and anti–PD-L1 agents in melanoma, lung cancer, and RCC. The mechanism of action of these agents is restarting antitumor immune responses; thus, the AE profile likely results from stimulation of T cells.

For ipilimumab, these events typically include colitis/diarrhea, rash, hepatitis, endocrinopathies, and, less frequently, uveitis and nephritis. Regarding the frequency of these irAEs, a pooled analysis of completed ipilimumab clinical trials showed that 64% of patients experienced an AE of any grade that was attributed to an immune mechanism, and 18% of patients experienced an irAE of grade 3 or higher (Ibrahim, Berman, & de Pril, 2011).

Some AEs observed to date for anti–PD-1 and anti–PD-L1 agents also appear to be immune-related and may overlap with the ipilimumab AE profile, including diarrhea, rash, pruritus, and endocrinopathies (Figure 2; Wolchok et al., 2013; Brahmer et al., 2013; Topalian et al., 2012; Hamid et al., 2013; Garon et al., 2013). However, based on initial reports, the incidence of irAEs with these agents may occur less frequently than with ipilimumab, and some types of irAEs may differ. As previously mentioned, although AEs such as diarrhea, nausea, and fatigue caused by chemotherapy or immunotherapy may appear similar, the pathogenic mechanism of action is very different, and this may affect how the AEs are managed.

An important example of this phenomenon is diarrhea, which can occur with cytotoxic therapies, targeted therapies, and immune checkpoint inhibitors. Chemotherapy can cause the death of rapidly dividing intestinal epithelial cells, leading to intestinal mucosal damage (including loss of intestinal epithelium, superficial necrosis, and inflammation of the bowel wall) and an imbalance between absorption and secretion in the small bowel (Florea & Büsselberg, 2011; Stein et al., 2010). Current treatment for grade 1–2 chemotherapy-induced diarrhea is diet modification and loperamide therapy, or use of other over-the-counter diarrheal medication, until resolution (Stein, Voigt, & Jordan, 2010).

In contrast, diarrhea with immunotherapy is likely due to an immune reaction to gut-associated or self antigens (Fecher, Agarwala, Hodi, & Weber, 2013). This type of diarrhea can escalate quickly, can become self-perpetuating, and may lead to tissue destruction and gut perforation if not treated promptly (Fecher et al., 2013).

For patients on ipilimumab who experience moderate diarrhea (4 to 6 stools a day over baseline), over-the-counter remedies may be helpful. However, any patient with more severe diarrhea or persistent or worsening symptoms should be started on corticosteroids. A failure to recognize diarrhea as potentially immune-related or to manage with proper treatment could escalate the event to a life-threatening situation. Thus, diarrhea in any patient receiving ipilimumab should be thought to be related to the drug’s immune activation unless another etiology is known (Fecher et al., 2013; Weber, Kahler, & Hauschild, 2012). As such, patients receiving ipilimumab, caregivers, and APs must take diarrhea very seriously.

Disorders of endocrine function have been reported with immune checkpoint inhibitors and are not typically seen with chemotherapies. In clinical trials of immune checkpoint inhibitors, reported endocrinopathies included hypothyroidism, hyperthyroidism, hypopituitarism, hypophysitis, thyroiditis, and adrenal insufficiency, with frequencies 8% (Topalian et al., 2012; Brahmer et al., 2013; Hamid et al., 2013; Hodi et al., 2010). As performed in clinical trials, regular monitoring of thyroid levels and use of replacement hormones, if needed, may be important management strategies for patients treated with ipilimumab or other immune checkpoint inhibitors (as these drugs become available in the clinic). Some endocrine disorders are persistent and may require long-term hormone replacement.

**Figure 3:**
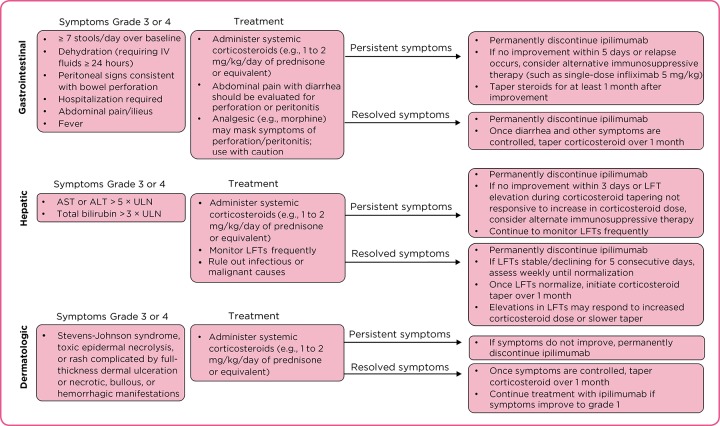
Management strategy guidelines for immune-related adverse events associated with ipilimumab. ALT = alanine transaminase; AST = aspartate transaminase; LFT = liver function tests; ULN = upper limit of normal (Fecher et al., 2013; Weber et al., 2012).

For ipilimumab-treated patients, guidelines outlining management strategies for AEs suspected to be immunologic have been developed (Fecher et al., 2013; Weber et al., 2012). They recommend that liver enzyme and thyroid hormone levels be evaluated prior to each ipilimumab dose. Also, they emphasize the use of steroids to manage irAEs and provide guidance on when to withhold or permanently discontinue ipilimumab or to escalate to the use of other agents, e.g., alternative immunosuppressants (Figure 3). Similar approaches to AE management were used in clinical trials of anti–PD-1 pathway agents and resulted in successful resolution of AEs in most cases (Brahmer et al., 2013; Topalian et al., 2012; Wolchok et al., 2013). However, whether these approaches are relevant to other immune checkpoint inhibitors is as yet unknown and will be clarified upon availability of more safety data from ongoing clinical trials of these agents.

Pneumonitis has been observed with anti–PD-1/PD-L1 agents. Although no grade 3–5 pneumonitis has been reported for pembrolizumab, pidilizumab, or MPDL3280A at the time of writing, three deaths due to pneumonitis occurred in nivolumab-treated patients early in the phase I trial (Topalian et al., 2012; Garon et al., 2013; Hamid et al., 2013; Horn et al., 2013; Westin et al., 2012).

Thus, new or worsening respiratory symptoms must be evaluated promptly in patients who have received immune checkpoint inhibitors. This may be challenging in patients with lung cancer who have underlying respiratory issues. In addition to vigilance and a multidisciplinary approach, pneumonitis associated with immune checkpoint inhibitors has been managed with drug discontinuation, corticosteroids, and use of other immunosuppressive agents as needed (Topalian et al., 2012; Topalian et al., 2014). One report described complete resolution of two cases of grade 3 nivolumab-associated pneumonitis after protracted prednisone tapers from 120 mg over 2 to 4 months (Weber et al., 2013).

Advanced practitioners play a key role in assessing and addressing symptoms during each office visit and follow-up call. The failure to identify and promptly treat irAEs early or poor patient compliance with steroid treatment can lead to more serious events (Hodi et al., 2010; Rubin, 2012; Andrews & Holden, 2012; Weber et al., 2012). In the pivotal ipilimumab studies, the median time to resolution of irAEs ranged from 4.9 to 9.9 weeks (Hodi et al., 2010; Robert et al., 2011). Time to recovery may be expedited when patients, caregivers, and nurses promptly report early signs of irAEs and the events are effectively managed (Fecher et al., 2013).

**Figure 4:**
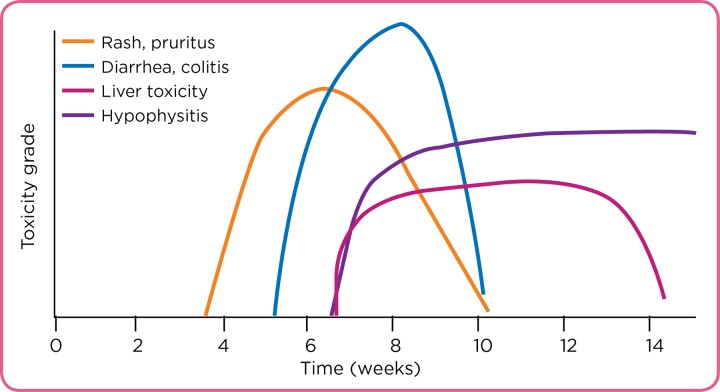
Relative time course for the appearance of different immune-related adverse events reported for ipilimumab, with rash and diarrhea occurring first after treatment initiation, followed by liver or endocrine toxicities, usually of a lower grade. Reprinted with permission. © 2012 American Society of Clinical Oncology. All rights reserved. Weber JS et al. J Clin Oncol. 2012; 30(21):2691–2697.

Timing of irAEs may also be related to the immunomodulating mechanism of action. In the case of ipilimumab, a majority of irAEs initially manifest during receipt of the first four doses of the agent; however, delayed AEs may occur weeks to months after initiation of ipilimumab (Fecher et al., 2013). Although individual patient experiences will vary, a relative time course for the appearance of different irAEs has been described for ipilimumab, with rash and diarrhea occurring first after treatment initiation, followed by liver or endocrine toxicities, usually of a lower grade (Figure 4).

## THE ROLE OF THE AP

It is crucial for APs in the clinic to be educated and proactive when treating patients receiving immuno-oncology agents. Nurses in clinics with extensive ipilimumab experience have developed processes to educate patients and caregivers, monitor patients during therapy, assess patients for drug-related AEs, and aid in AE management (Rubin, 2012; Andrews & Holden, 2012). To reduce psychological distress, patients starting ipilimumab should be informed as to what to expect prior to the first infusion and at every point of contact. Patient-friendly literature should be provided, including basic information on the immune system and immune-targeting therapy.

In one nurse-authored report, the drug’s mechanism of action is described in a simplified format using the "gas and brake pedal" analogy (Ledezma & Heng, 2013). In this description, pressing the gas pedal is analogous to restoring T-cell activity and necessary for a car to move forward (i.e., starting an immune response against the tumor). However, a car uses the brake pedal to prevent unwanted speed. Similarly, the body uses immune checkpoint pathways to prevent uncontrolled and potentially damaging immune responses. Immune checkpoint inhibitors "lift the foot off the brake," so the car can continue forward (i.e., so that a T-cell–mediated antitumor immune response can continue). This analogy may also explain the irAEs seen with immunotherapy drugs, where immune responses against healthy tissues that usually would be stopped are not as tightly controlled.

Setting patient expectations ahead of treatment is key to reducing patient anxiety later. As part of their education, patients should be informed that, despite apparent disease progression (enlarging lesions) after starting ipilimumab, they ultimately may respond to and benefit from treatment. The immune-mediated mechanism of action of ipilimumab can also be used to explain why the first scanning period is not until week 12, unlike with chemotherapy, as it may take time for an effective antitumor immune response to become apparent. Clinical assessment is also likely to be delayed for PD-1/PD-L1–blocking agents as well, although early evidence suggests that clinical responses with PD-1–pathway agents may occur more quickly than with ipilimumab.

Finally, patients should be told how important it is for them to be certain that anyone who cares for them—e.g., a doctor in a local emergency department or their primary care doctor—should be aware that they have received immunotherapy, as it may impact treatment decisions, particularly concerning irAEs. Patients receiving ipilimumab may receive a wallet card containing important AE and health-care provider information.

## SUMMARY

Ipilimumab has provided hope to patients with metastatic melanoma and their health-care providers. Immunotherapy has spurred new excitement and ongoing research into a number of other immune checkpoint blockade therapies, including agents that block the PD-1 pathway. Initial data have provided evidence that immune checkpoint inhibitors may become treatment options for patients with different cancers, including melanoma, lung cancer, prostate cancer, RCC, mesothelioma, and hematologic malignancies. Early evidence also suggested that combinations of more than one immune checkpoint inhibitor may provide higher response rates, quicker responses, and more sustained antitumor responses than single-agent treatment. APs who familiarize themselves with immunotherapy—including how to identify the signs and symptoms of adverse reactions, manage these events, and best educate patients and caregivers—will be well positioned as these therapies become more common in the clinic.

**Acknowledgments**

The author takes full responsibility for the content of this publication and confirms that it reflects her viewpoint and medical expertise. She also wishes to acknowledge StemScientific, funded by Bristol-Myers Squibb, for providing writing and editorial support. Neither Bristol-Myers Squibb nor StemScientific influenced the content of the manuscript, nor did the author receive financial compensation for authoring the manuscript.
